# A Multiplex PCR Detection Assay for the Identification of Clinically Relevant *Anaplasma* Species in Field Blood Samples

**DOI:** 10.3389/fmicb.2020.00606

**Published:** 2020-04-07

**Authors:** Yongshuai Peng, Shanshan Zhao, Kunlun Wang, Jinxing Song, Yaqun Yan, Yongchun Zhou, Ke Shi, Fuchun Jian, Rongjun Wang, Longxian Zhang, Changshen Ning

**Affiliations:** ^1^College of Animal Science and Veterinary Medicine, Henan Agricultural University, Zhengzhou, China; ^2^College of Animal Medical Science, Henan University of Animal Husbandry and Economy, Zhengzhou, China; ^3^International Joint Research Laboratory for Zoonotic Diseases of Henan, Zhengzhou, China

**Keywords:** *Anaplasma capra*, *Anaplasma bovis*, *Anaplasma ovis*, *Anaplasma phagocytophilum*, multiplex PCR

## Abstract

The genus *Anaplasma* (Rickettsiales: Anaplasmataceae), which includes the species *Anaplasma capra*, *Anaplasma bovis*, *Anaplasma ovis*, and *Anaplasma phagocytophilum*, is responsible for a wide variety of infections in both human and veterinary health worldwide. Multiple infections with these four *Anaplasma* pathogens have been reported in many cases. We introduce a novel multiplex PCR for the simultaneous detection of *A. capra*, *A. bovis*, *A. ovis*, and *A. phagocytophilum*, based on species-specific primers against the *groEL* (*A. capra* and *A. bovis*), *msp4* (*A. ovis*), and 16S rRNA (*A. phagocytophilum*) genes. To verify the specificity of the PCR reactions, we evaluated four sets of primers to analyze samples containing different blood pathogens. The sensitivity of the multiplex PCR was evaluated by amplifying 10-fold dilutions of total genomic DNA extracted from sheep blood infected with *A. capra*, *A. bovis*, *A. ovis*, or *A. phagocytophilum*. The reproducibility of the assay was evaluated by testing 10-fold dilutions of total genomic DNA extracted from sheep blood infected with these pathogens from 10^0^ to 10^–3^ ng/μL per reaction in triplicate on three different days. A total of 175 field blood DNA samples were used to evaluate the reproducibility of multiplex PCR compared with the simplex PCRs. PCR primers used in this study were confirmed to be 100% species-specific using blood pathogens previously identified by other methods. The lower limit of detection of the multiplex PCR with good repeatability enabled the detection of *A. capra*, *A. bovis*, *A. ovis* and *A. phagocytophilum* at concentrations of 3 × 10^–5^, 5 × 10^–7^, 2 × 10^–5^, and 7 × 10^–7^ ng/μL, respectively. There was no significant difference between conventional and multiplex PCR protocols used to detect the four *Anaplasma* species (*P* > 0.05). The results of the multiplex PCR revealed that the *A. capra groEL* gene, the *A. bovis groEL* gene, the *A. ovis msp4* gene, and the *A. phagocytophilum* 16S rRNA gene were reliable target genes for species identification in clinical isolates, being specific for each of the four target *Anaplasma* species. Our study provides an effective, sensitive, specific, and accurate tool for the rapid differential clinical diagnosis and epidemiological surveillance of *Anaplasma* pathogens in sheep and goats.

## Introduction

The genus *Anaplasma* (Rickettsiales: Anaplasmataceae) comprises tick-transmitted obligate intracellular bacterial species including *Anaplasma capra*, *Anaplasma bovis*, *Anaplasma ovis*, and *Anaplasma phagocytophilum*, which are responsible for a wide variety of different infections in both human and veterinary health (Guo et al., 2018; Kundave et al., 2018; Guimarães et al., 2019). *A. capra* is a potential novel tick-borne *Anaplasma* species, which was identified in goats in China (Liu et al., 2012) and provisionally nominated it “*Anaplasma capra*” as the causative agent of human infections as reported by Li et al. (2015). This pathogen may be responsible for anaplasmosis cases and may be a substantial public health concern; it also appears to be widely distributed in China and South Korea (Li et al., 2015; Peng et al., 2018; Yang et al., 2018a). *A. bovis* infects monocytes and tissue macrophages of small mammals and ruminants (Dumler et al., 2001). The DNA of this pathogen has been detected in cattle, goats, sheep, dogs, and some small wild mammals (Fukui and Inokuma, 2019; Giglioti et al., 2019; Yang et al., 2019). It can cause a variety of clinical symptoms, including weight loss, fever, anemia, listlessness, and death in some cases (Aktas and zübek, 2015). *A. ovis* is an intra-erythrocytic rickettsial pathogen that mainly affects domestic sheep and goats, but has also been reported to be present in some wild ruminants and dogs with mild clinical symptoms (Zaid et al., 2019). However, symptoms may be exacerbated by stressors such as co-infection, elevated temperature, and animal movement disorders throughout Asia, Africa, Europe and the United States (Hornok et al., 2011; Cabezas-Cruz et al., 2019; Enkhtaivan et al., 2019; Liu et al., 2019). This pathogen also poses a potential threat to humans (Wei et al., 2017). The first human case of *A. ovis* infection was reported in Cyprus and was characterized by a fever, hepatosplenomegaly, and lymphadenopathy (Chochlakis et al., 2010). *A. phagocytophilum* mainly affects small ruminants in Hungary, Romania, Italy, Kenya, South Africa, China, Vietnam, Morocco, and Mexico (Ait Lbacha et al., 2017; Seo et al., 2018a; Han et al., 2019; Hornok et al., 2019), but can also cause human granulocytic anaplasmosis. The pathogen infects neutrophils and survives by inhibiting or delaying important antimicrobial mechanisms in host cells (Goel et al., 2018; Lee et al., 2018; Dehhaghi et al., 2019). Simultaneous infection of these four *Anaplasma* agents or mixed infections by different *Anaplasma* species has also been reported (Halajian et al., 2018; Wang et al., 2018; Seo et al., 2018b).

Traditionally, laboratory detection of *Anaplasma* species in animals or humans was dependent on the microscopic examination of blood samples (Giglioti et al., 2019). However, using this method, it was difficult to clearly distinguish between *A. capra*, *A. bovis*, *A. ovis*, and *A. phagocytophilum*, as they are highly similar in terms of their morphological characteristics (Liu et al., 2012; Li et al., 2015; Wei et al., 2017; Lee et al., 2018). It was also difficult to distinguish these agents from other blood pathogenic agents, such as members of the *Theileria* and *Babesia* genera (Al-Hosary et al., 2018; Pradeep et al., 2019). Especially when the pathogen load is low, it is difficult to distinguish between these pathogens by microscopic examination. In recent years, molecular methods have been increasingly used in microbiology laboratories, such as duplex PCR based assays and loop-mediated isothermal amplification assays, as well as other recently developed methods that could lead to the development of new diagnostic tests to identify different *Anaplasma* species (Cui et al., 2017; Yang et al., 2018a; Giglioti et al., 2019). The development of multiplex PCR can enable positive detection without the need for additional reagents or increased amounts of input DNA (Hao et al., 2019). However, primer design for multiplex PCR becomes more challenging when the number of PCR reactions increases. Hence, to the best of our knowledge, no multiplex PCR assay for the identification of clinically relevant *Anaplasma* species in field blood samples has previously been reported.

In this study, we developed an accurate, specific and sensitive multiplex PCR, using a combination of specially designed primers and previously reported primers, for the identification of *Anaplasma* species in field blood samples from animals or patients suspected of being infected with members of this genus.

## Materials and Methods

### Control DNA Samples

Preserved DNA of *Anaplasma* spp. (*A. capra*, *A. bovis*, *A. ovis*, *A. phagocytophilum*) was used as positive controls in this study. DNA samples positive for *Anaplasma marginale*, *Anaplasma platys*, *Theileria ovis*, *Theileria annulata*, *Theileria uilenbergi*, *Theileria luwenshuni*, *Babesia motasi*, and *Toxoplasma gondii* were used as negative controls. *T. ovis* and *T. uilenbergi* positive samples were provided by Professor Mengqi from Tarim University and the others were maintained at −80°C at the Laboratory of Parasitology of Henan Agricultural University. All the control DNA samples were evaluated with a spectrophotometer (Nanodrop One^c^; Thermo Fisher Scientific, Wilmington, DE, United States) prior to use to ensure that the concentration of DNA samples used in the test were >20 ng/μL and verified it by specific primers before use. Double-distilled (dd) H_2_O was used as a blank control.

### DNA Extraction

DNA of field samples was extracted from 250 μL of blood using the Blood DNA Kit (OMEGA Bio-Tek, Norcross, GA, United States), in accordance with the manufacturer’s instructions. The DNA of each sample was eluted in 200 μL of elution buffer. All DNA samples were examined using the spectrophotometer to test their quality and quantity, and were stored at −20°C until use.

### Design of PCR Primers Used for the Detection of Target *Anaplasma* spp.

Primers were designed against regions of the *A. bovis groEL* gene and the *A. phagocytophilum* 16S rRNA gene using Premier 5 (Premier Biosoft International, Palo Alto, CA, United States). These regions were identified through alignments of nucleotide sequences obtained in this study and sequences available from the GenBank database (KX987399, KU585932, and KY425449 for *A. bovis*; KY242452, KR002115, LC060987, KF569915, and KC916737 for *A. phagocytophilum*). The specificity of each primer set was evaluated using the Basic Local Alignment Search Tool (BLAST) from the National Center for Biotechnology Information (NCBI) database^1^. For the detection of *A. capra* and *A. ovis* targeting the *groEL* and *msp4* genes, respectively, previously described primers were used (Torina et al., 2012; Yang et al., 2016). The primers are described in Table 1. The primer sets were synthesized by Sangon Biotech Company (Shanghai, China).

**TABLE 1:** List of the primers used in the PCR assays in this study.

**Target organisms**	**The primer sets used in the multiplex PCR**				**The primer sets used in the conventional PCR**			
	
	**Target gene**	**Primer sequence (5′-3′)**	**Amplicon size (bp)**	**References**	**Target gene**	**Primer sequence (5′-3′)**	**Amplicon size (bp)**	**References**
*A. capra*	*groEL*	TGAAGAGCATCAAACCCGAAG	874	Yang et al. (2016)	*msp4*	GGGTTCTGATATGGCATCTTC	656	Li et al. (2015)
		CTGCTCGTGATGCTATCGG				GGGAAATGTCCTTATAGGATTCG		
*A. bovis*	*groEL*	GTGGGATGTACTGCTGGACC	529	This study	16S rRNA	CTCGTAGCTTGCTATGAGAAC	551	Kawahara et al. (2006)
		ATGGGGAGAGATATCCGCGA				TCTCCCGGACTCCAGTCTG		
*A. ovis*	*msp4*	TGAAGGGAGCGGGGTCATGGG	347	Torina et al. (2012)	*msp4*	CCGGATCCTTAGCTGAACAGGAATCTTGC	845	de la Fuente et al. (2007)
		GAGTAATTGCAGCCAGGCACTCT				GGGAGCTCCTATGAATTACAGAGAATTGTTTAC		
*A. phagocytophilum*	16S rRNA	AGTGCTGAATGTGGGGATAATTTATCTCCGTG	172	This study	16S rRNA	GCTGAATGTGGGGATAATTTAT	641	Kawahara et al. (2006)
		CTAATCTCCATGTCAAGGAGTGGTAAGGTTT				ATGGCTGCTTCCTTTCGGTTA		

### Optimization of the PCR Conditions

To ensure optimal amplification conditions for the four target sequences in a multiplex PCR assay, a range of PCR-related parameters were evaluated such as the annealing temperatures of the primers, the dosage of primers, and the concentrations of La *Taq* DNA polymerase. To examine these parameters, a number of tests were performed in 25 μL reaction volumes.

PCR conditions were investigated including the variation of the annealing temperature from 57 to 64°C, the concentration of primer sets from 0.08 to 0.56 μM, the concentration of La Taq DNA polymerase (TaKaRa, Dalian, China) from 0.75 to 1.75 U, and the concentration of PCR buffer (10×) and the dNTPs (2.5 mM) from 1.5 to 3 μL and 2 to 6 μL, respectively. The optimization of the multiplex PCR conditions was based on a previous report by Henegariu et al. (1997).

### Plasmid Construction

For the construction of plasmids containing the *A. capra groEL* gene, the *A. bovis groEL* gene, the *A. ovis msp4* gene, and the *A. phagocytophilum* 16S rRNA gene, the corresponding PCR products of 874, 529, 347 and 172 bp were cloned into pMD-18T (TaKaRa) and then propagated in *Escherichia coli* DH5α competent cells (Sangon Biotech). Plasmid DNA was purified from transformed cells using the SanPrep Column Plasmid Mini-Prep Kit (Sangon Biotech) and quantified using a spectrophotometer (Nanodrop One^c^; Thermo Fisher Scientific).

To generate standard curves for quantitative determinations and to assess the amplification efficiency, plasmids were 10-fold-diluted in elution buffer (Sangon Biotech), representing 10^0^–10^–4^ ng/μL DNA template. Aliquots of each dilution were frozen at −20°C until use. To minimize the potential for contamination, the standard plasmid DNA was stored in a separate laboratory.

### Evaluation of the Specificity, Sensitivity and Reproducibility of the Multiplex PCR

To rule out the possibility of cross-reactions with other closely related pathogens of the blood, the specificity of the multiplex PCR was evaluated by testing positive control DNA from blood samples infected with *A. capra*, *A. bovis*, *A. ovis*, *A. phagocytophilum*, *A. marginale*, *A. platys*, *Theileria ovis*, *T. annulata*, *T. uilenbergi*, *T. luwenshuni*, *B. motasi*, and *T. gondii*. The negative control was ddH_2_O, and 29.4 ng/μL was the lowest DNA concentration used in the assay.

Ten-fold dilutions of total genomic DNA extracted from the blood of sheep infected with *A. capra*, *A. bovis*, *A. ovis*, and *A. phagocytophilum* were used to evaluate the sensitivity of the multiplex PCR. Each experiment consisted of triplicate tests on two replicates.

The reproducibility of the assay was evaluated by testing 10-fold dilutions of total genomic DNA extracted from sheep blood infected with *A. capra*, *A. bovis*, *A. ovis*, and *A. phagocytophilum* from 10^0^ to 10^–3^ ng/μL per reaction in triplicate on three different days. Kendall’s coefficient of concordance (Kendall’s W) was calculated to evaluate the repeatability of the method. Its value can be between 0 and 1; the higher the value, the better the repeatability.

### Quantification of Pathogens in Clinical Samples

To quantify the pathogen DNA of *A. capra*, *A. bovis*, *A. ovis*, and *A. phagocytophilum* in clinical samples, the qTOWER3 G QPCR System (Analytikjena Technologies, Jena, Germany) was used for the real-time PCR assay performed in a final volume of 10 μL containing 5 μL of TB Green^TM^ Premix Ex Taq^TM^ (TaKaRa, Dalian, China), 0.4 μM of each primer and 2 μL of the DNA template. The thermal profile of the PCR was as follows: 30 s at 95°C for denaturation, followed by 40 cycles of denaturation at 95°C for 5 s, annealing at 63°C for 30 s, and extension at 72°C for 45 s. The final extension step was 1 min at 72°C.

### Application to Clinical Samples

A total of 175 blood samples (sheep 99, goats 76) were collected at different time points from a simplex farm in Linyou County, Shanxi Province. The sheep and goats in this farm were grazed perennially and highly infested with ticks. DNA of these samples was used to evaluate the efficiency of the simplex and multiplex PCR protocols. Conventional PCR conditions as previously described were performed and compared in terms of efficiency and capability to the multiplex PCR. The used primer sets are shown in Table 1. Upon detecting *Anaplasma* in field samples by the multiplex PCR, all positive PCR products were sent to a sequencing company for sequencing, and the results were consistent with expectations. Infection rates were assessed using the Chi-square test with Yates’ correction, and a *P*-value ≤0.05 was considered to represent statistical significance.

## Results

### Optimization of the Multiplex PCR

**FIGURE 1:**
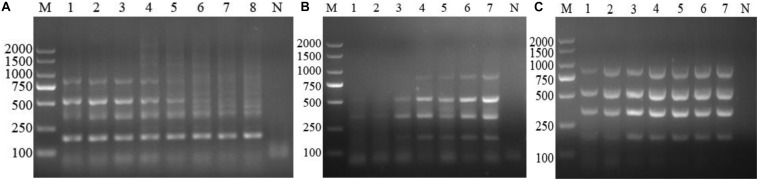
Optimization of the multiplex PCR components. **(A)** Annealing temperature gradients, M: DL2000 marker. Lanes 1–8: 64, 63, 62, 61, 60, 59, 58, and 57°C, respectively. **(B)** Dose of *Anaplasma* primers, lane M: DL2000 marker; Lanes 1–7: 0.08, 0.16, 0.24, 0.32, 0.4, 0.48, and 0.56 μM, respectively; Lane N: negative control. **(C)** La Taq DNA polymerase. M: DL2000 marker; Lanes 1–7: 0.75, 1.0, 1.25, 1.5, 1.75, 2, and 2.25 U, respectively; Lane N: negative control.

After optimization, the optimum multiplex PCR assay was performed in a final volume of 25 μL, containing 2.5 μL of 10× PCR La buffer, 4 μL of dNTPs at 2.5 mM, 1.25 U of La *Taq* DNA polymerase, 0.32 μM of each primer, and 2 μL of the DNA template. The thermal profile of the PCR was as follows: 5 min at 94°C for denaturation, followed by 35 cycles of denaturation at 94°C for 30 s, annealing at 63°C for 30 s, and extension at 72°C for 1 min. The final extension step was 10 min at 72°C. The PCR products (5 μL) were analyzed using a UV gel imaging system following electrophoresis in a 1.5% agarose gel and staining with DNA GREEN (Solarbio, Beijing, China) (Figures 1A–C).

### Primer Specificity

**FIGURE 2:**
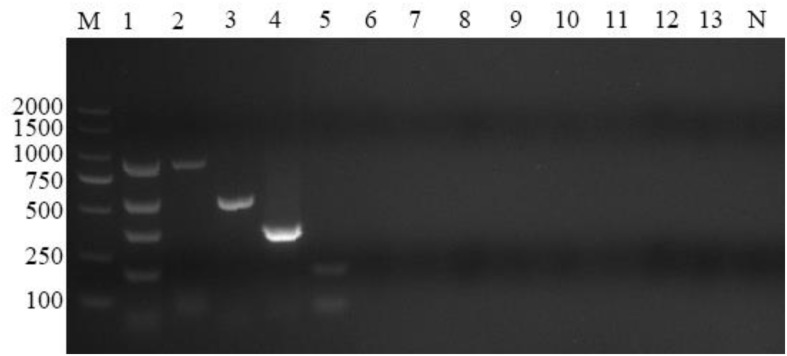
Multiplex PCR specificity test. Four primer sets could amplify genus-specific bands of 874, 529, 347, and 172 bp only in samples positive for *Anaplasma capra*, *Anaplasma bovis*, *Anaplasma ovis*, and *Anaplasma phagocytophilum*, respectively. M: DL2000 marker; Lane 1: mixed DNA samples from the four *Anaplasma* species; Lanes 2–13: samples positive for *A. capra*, *A. bovis*, *A. ovis*, *A. phagocytophilum*, *A. marginale*, *A. platys*, *Theileria ovis*, *Theileria annulata*, *Theileria uilenbergi*, *Theileria luwenshuni*, *Babesia motasi*, and *Toxoplasma gondii*, respectively; Lane N: negative control.

To characterize the specificity of the reactions, the four sets of primers were evaluated using samples containing *A. capra*, *A. bovis*, *A. ovis*, *A. phagocytophilum*, *A. marginale*, *A. platys*, *Theileria ovis*, *T. annulata*, *T. uilenbergi*, *T. luwenshun*, *B. motasi*, or *T. gondii* DNA, and ddH_2_O was used as a blank control. Fragments of the expected sizes were generated from the positive DNA templates. Using the mixed DNA of the four *Anaplasma* species as templates, four bands of expected sizes were observed, and the target bands for infection with a simplex *Anaplasma* agent appeared separately. No amplification signals were detected with the negative control (*A. marginale*, *A. platys*, *Theileria ovis*, *T. annulata*, *T. uilenbergi*, *T. luwenshuni*, *B. motasi*, and *T. gondii*) and blank control templates (Figure 2). The results showed that the reaction products could be distinguished by gel electrophoresis and the primer sets used in this study were species-specific.

### Sensitivity of the Multiplex PCR

**FIGURE 3:**
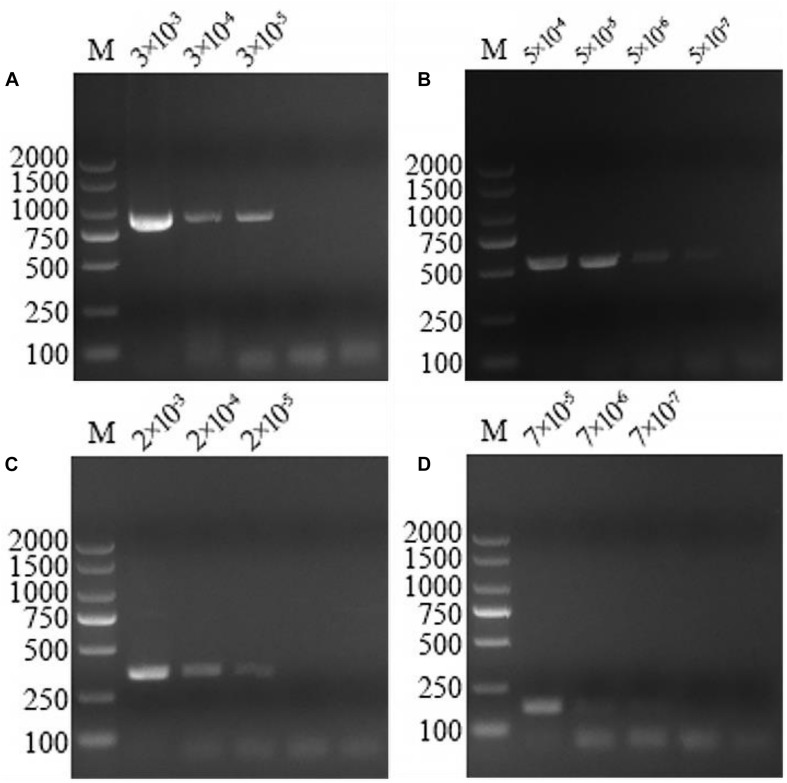
Multiplex PCR sensitivity tests on serially diluted positive DNA sample templates. At the top of the figure is dilution factor, with a unit of ng/μL. The left and right sides of the figure show the molecular weight of the DNA. **(A)** The sensitivity of the multiplex PCR assay for *Anaplasma capra* was 3 × 10^–5^ ng/μL. **(B)** The sensitivity of the multiplex PCR assay for *Anaplasma bovis* was 5 × 10^–7^ ng/μL. **(C)** The sensitivity of the multiplex PCR assay for *Anaplasma ovis* was 2 × 10^–5^ ng/μL. **(D)** The sensitivity of the multiplex PCR assay for *Anaplasma phagocytophilum* was 7 × 10^–7^ ng/μL.

The optimized multiplex PCR was evaluated by amplifying 10-fold serial dilutions (10^0^–10^–4^ ng/μL) of the mixture DNA infected with these pathogens extracted from sheep blood. The lower limit of detection of the multiplex PCR could detect *A. capra*, *A. bovis*, *A. ovis*, and *A. phagocytophilum* at concentrations of 3 × 10^–5^, 5 × 10^–7^, 2 × 10^–5^, and 7 × 10^–7^ ng/μL, respectively (Figure 3).

### Repeatability of the Multiplex PCR

Kendall’s W values for inter-assay reproducibility were found to range from 0.80 to 0.90 (Supplementary Appendix). This shows that the optimized multiplex PCR has good repeatability.

### Application to Clinical Samples

**FIGURE 4:**
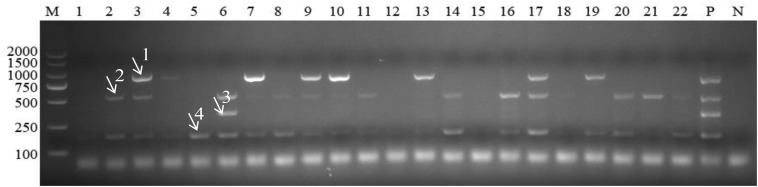
Detection of *Anaplasma* in field samples using multiplex PCR. M: DL2000 marker; Lanes 1–22: field samples; P: positive control; N: negative control. Arrows with numbers (1–4) indicate the amplicons of *Anaplasma capra* 874 bp, *Anaplasma bovis* 529 bp, *Anaplasma ovis* 347 bp, and *Anaplasma phagocytophilum* 172 bp.

Out of a total of 175 samples, multiplex PCR revealed 41 (23.4%), 68 (38.9%), 33 (18.9%), and 105 (60.0%) blood samples positive for *A. capra*, *A. bovis*, *A. ovis*, and *A. phagocytophilum*, respectively. By comparison, the positive detection rates of these four *Anaplasma* species using simplex PCR and conventional PCR as previously described were 25.1, 47.4, 20.0, and 60.6, and 24.0, 44.6, 20.6, and 58.3%, respectively (Table 2). There was no significant difference among the three methods used to detect the four *Anaplasma* species (*P* > 0.05). An example of the multiplex PCR results for the detection of *Anaplasma* spp. in field samples is provided in Figure 4.

**TABLE 2:** Simplex PCR, multiplex PCR, and conventional PCR testing of field blood samples.

**Target organisms**	**The positive rate of field samples (%)**		
	
	**Simplex PCR**	**Multiplex-PCR**	**Conventional PCR**
*A. capra*	25.1% (44/175)	23.4% (41/175)	24.0% (42/175)
*A. bovis*	47.4% (83/175)	38.9% (68/175)	44.6% (78/175)
*A. ovis*	20.0% (35/175)	18.9% (33/175)	20.6% (36/175)
*A. phagocytophilum*	60.6% (106/175)	60.0% (105/175)	58.3% (102/175)

## Discussion

*Anaplasma* species are some of the most widespread microbes with potential human and animal pathogenicity that are transmitted by ticks (Kim et al., 2014; Sun et al., 2015; Ben Said et al., 2018). Among these, *A. capra*, *A. phagocytophilum*, and *A. ovis* are tick-borne zoonotic pathogens that are significant for public health (Chochlakis et al., 2010; Li et al., 2015; Lee et al., 2018), they have been detected in many domestic and wild animals across the world (Kawahara et al., 2006; Seo et al., 2018b; Yang et al., 2018b; Cabezas-Cruz et al., 2019). Additionally, recent reports have presented evidence of co-infection with two or more *Anaplasma* species in cattle, sheep, goats, and ixodid ticks worldwide (Rjeibi et al., 2018; Seo et al., 2018b; Wang et al., 2018; Zhou et al., 2018; Han et al., 2019). It is thus important to detect several *Anaplasma* species in a single DNA sample by using a single PCR reaction. This would also reduce the cost of the analysis, which is important particular in developing countries.

PCR assays have been used to sensitively and specifically detect *Anaplasma* species in many diagnostic laboratories. In fact, numerous reports have been published on the PCR detection of simplex *Anaplasma* species in infected animals and humans (Torina et al., 2012; Chi et al., 2013; Lee et al., 2018). Duplex PCR assays for the detection of co-infection with *A. marginale* (targeting the *msp4* gene) and *A. phagocytophilum* (targeting the *msp2* gene) in cattle (M’ghirbi et al., 2016) and duplex real-time PCR assays for the detection of co-infection with *A. marginale* (targeting the *msp1β* gene) and *Anaplasma centrale* (targeting the *groEL* gene) in sheep (Decaro et al., 2008) were previously reported. However, no previous study has developed an efficient and convenient PCR assay that would be more convenient and efficient for field diagnosis than the simplex or duplex PCR assays to detect multiple *Anaplasma* agents. Early diagnosis and treatment are required to reduce the morbidity and mortality associated with these diseases worldwide (Zhuo et al., 2019). Hence the present multiplex PCR assay could support the need for epidemiological assessments of the global distribution of *A. capra*, *A. bovis*, *A. ovis*, and *A. phagocytophilum* in animals and humans.

Several molecular techniques have been proposed for detecting and characterizing species belonging to the *Anaplasma* genus. Previous studies indicated that the 16S rRNA, 18S rRNA, *groEL*, *ankA*, citrate synthase (*gltA*), and major surface protein (*msps*) sequences are highly conserved in *Anaplasma* spp.; thus, they have been used widely to classify the *Anaplasma* genus (Lew et al., 2003; Lee et al., 2018; Song et al., 2018; Cabezas-Cruz et al., 2019). Currently, epidemiological investigation of *A. capra* uses an assay based on the 16S rRNA, *gltA* and *msp4* genes (Yang et al., 2018a). Molecular detection of *A. capra*, *A. bovis*, *A. ovis*, and *A. phagocytophilum* in infected ixodid ticks was based on the 16S rRNA, *gltA*, *msp4*, and 16S rRNA genes, respectively (Han et al., 2019). Additionally, molecular evidence of coinfection of *Anaplasma* species in small ruminants was obtained by targeting the 16S rRNA and *msp4* genes of *A. bovis*, *A. ovis*, and *A. phagocytophilum* (Yang et al., 2019). Here, we developed a multiplex PCR assay, which was able to specifically detect *A. capra*, *A. bovis*, *A. ovis*, and *A. phagocytophilum* from field samples. These detection limits of *A. bovis* and *A. phagocytophilum* were higher than those reported for the detection of *Anaplasma* spp. in a duplex PCR assay by Cui et al. (2017).

This method had one limitation. *A. capra* and *A. ovis* have slightly lower detection limits. The primers used to amplify *A. capra* and *A. ovis* thus need further improvement. Considering the convenience and high efficiency of this method, it may still be suitable for the detection and epidemiological investigation of *Anaplasma* in humans, animals and vector ticks.

## Conclusion

The results of the multiplex PCR revealed that the *A. capra groEL* gene, the *A. bovis groEL* gene, the *A. ovis msp4* gene, and the *A. phagocytophilum* 16S rRNA gene are reliable target genes for species identification in clinical isolates, as they are specific for each of the four target *Anaplasma* species. Other, closely related pathogens of blood tested negative in this PCR assay. In conclusion, our study provides an effective, sensitive, specific, and accurate tool for the rapid differential clinical diagnosis and epidemiological surveillance of *Anaplasma* pathogens (*A. capra*, *A. bovis*, *A. ovis*, and *A. phagocytophilum*) in sheep and goats.

## Data Availability Statement

All datasets generated for this study are included in the article/Supplementary Material.

## EthicS Statement

This research was conducted in accordance with the Chinese Laboratory Animal Administration Act (1988) after review and approval of its protocol by the Research Ethics Committee of Henan Agricultural University. Appropriate permission was obtained from the farm owners before the collection of blood specimens from their sheep and goats.

## Author Contributions

YP analyzed the samples, interpreted data, and wrote the manuscript. SZ conducted the laboratory examination of samples and sequences analysis. SZ, KW, JS, YY, YZ, and KS collected the samples. FJ, RW, LZ, and CN revised the manuscript, designed and supervised the study. All authors read and approved the final version of the manuscript.

## Conflict of Interest

The authors declare that the research was conducted in the absence of any commercial or financial relationships that could be construed as a potential conflict of interest.

## Footnotes

1 https://blast.ncbi.nlm.nih.gov/

## References

[B1] Ait Lbacha H. Alali S. Zouagui Z. El Mamoun L. Rhalem A. Petit E. (2017). High prevalence of *Anaplasma* spp. in small ruminants in morocco. *Transbound Emerg. Dis.* 64 250–263. 10.1111/tbed.12366 25916245

[B2] Aktas M. Zübek S. (2015). Bovine anaplasmosis in Turkey: first laboratory confirmed clinical cases caused by *Anaplasma phagocytophilum*. *Vet. Microbiol.* 178 246–251. 10.1016/j.vetmic.2015.05.021 26051478

[B3] Al-Hosary A. Ahmed L. Ahmed J. Nijhof A. Clausen P. H. (2018). Epidemiological study on tropical theileriosis (*Theileria annulata* infection) in the Egyptian Oases with special reference to the molecular characterization of *Theileria* spp. *Ticks Tick Borne Dis.* 9 1489–1493. 10.1016/j.ttbdis.2018.07.008 30033328

[B4] Ben Said M. Belkahia H. Messadi L. (2018). *Anaplasma* spp. in North Africa: a review on molecular epidemiology, associated risk factors and genetic characteristics. *Ticks Tick Borne Dis.* 9 543–555. 10.1016/j.ttbdis.2018.01.003 29398602

[B5] Cabezas-Cruz A. Gallois M. Fontugne M. Allain E. Denoual M. Moutailler S. (2019). Epidemiology and genetic diversity of *Anaplasma ovis* in goats in Corsica. *France. Parasit Vectors.* 12:3. 10.1186/s13071-018-3269-7 30606253 PMC6318933

[B6] Chi Q. Liu Z. Li Y. Yang J. Chen Z. Yue C. (2013). Development of a real-time PCR assay for detection and quantification of *Anaplasma ovis* infection. *Transbound Emerg. Dis*. 60 (Suppl. 2), 119–124. 10.1111/tbed.12139 24589111

[B7] Chochlakis D. Ioannou I. Tselentis Y. Psaroulaki A. (2010). Human anaplasmosis and *Anaplasma ovis* variant. *Emerging Infect Dis.* 16 1031–1032. 10.3201/eid1606.090175 20507768 PMC3086243

[B8] Cui Y. Zhang Y. Jian F. Zhang L. Wang R. Cao S. (2017). Development of duplex PCR for simultaneous detection of *Theileria* spp. and Anaplasma spp. in sheep and goats. *Exp. Parasitol.* 176 1–7. 10.1016/j.exppara.2017.01.011 28153803

[B9] de la Fuente J. Atkinson M. W. Naranjo V. Fernandez de Mera I. G. Mangold A. J. Keating K. A. (2007). Sequence analysis of the msp4 gene of *Anaplasma ovis* strains. *Vet. Microbiol.* 119 375–381. 10.1016/j.vetmic.2006.09.011 17052866

[B10] Decaro N. Carelli G. Lorusso E. Lucente M. S. Greco G. Lorusso A. (2008). Duplex real-time polymerase chain reaction for simultaneous detection and quantification of *Anaplasma marginale* and *Anaplasma centrale*. *J. Vet. Diagn Invest.* 20 606–611. 10.1177/104063870802000511 18776093

[B11] Dehhaghi M. Kazemi Shariat Panahi H. Holmes E. C. Hudson B. J. Schloeffel R. Guillemin G. J. (2019). Human tick-borne diseases in Australia. *Front. Cell Infect. Microbiol.* 9:3. 10.3389/fcimb.2019.00003 30746341 PMC6360175

[B12] Dumler J. S. Barbet A. F. Bekker C. P. Dasch G. A. Palmer G. H. Ray S. C. (2001). Reorganization of genera in the families Rickettsiaceae and Anaplasmataceae in the order Rickettsiales: unification of some species of *Ehrlichia* with *Anaplasma*, *Cowdria* with *Ehrlichia* and *Ehrlichia* with *Neorickettsia*, descriptions of six new species combinations and designation of *Ehrlichia equi* and ‘HGE agent’ as subjective synonyms of *Ehrlichia phagocytophila*. *Int. J. Syst. Evol. Microbiol.* 51 2145–2165. 10.1099/00207713-51-6-2145 11760958

[B13] Enkhtaivan B. Narantsatsral S. Davaasuren B. Otgonsuren D. Amgalanbaatar T. Uuganbayar E. (2019). Molecular detection of *Anaplasma ovis* in small ruminants and ixodid ticks from Mongolia. *Parasitol. Int.* 69 47–53. 10.1016/j.parint.2018.11.004 30458297

[B14] Fukui Y. Inokuma H. (2019). Subclinical infections of *Anaplasma phagocytophilum* and *Anaplasma bovis* in dogs in Ibaraki. *Japan.Jpn. J. Infect. Dis.* 72 168–172. 10.7883/yoken.JJID.2018.470 30700657

[B15] Giglioti R. Bassetto C. C. Okino C. H. de Oliveira H. N. de Sena Oliveira M. C. (2019). Development of a loop-mediated isothermal amplification (LAMP) assay for the detection of *Anaplasma marginale*. *Exp. Appl. Acarol.* 77 65–72. 10.1007/s10493-018-0327-y 30478537

[B16] Goel R. Westblade L. F. Kessler D. A. Sfeir M. Slavinski S. Backenson B. (2018). Death from transfusion-transmitted *Anaplasmosis*. New York, USA, 2017. *Emerging Infect. Dis.* 24 1548–1550. 10.3201/eid2408.172048 PMC6056119 30016241

[B17] Guimarães A. Raimundo J. M. Peixoto M. P. da Silva C. B. Pires M. S. Santos H. A. (2019). Molecular detection, characterization of *Anaplasma* spp. in domestic cats from Rio de Janeiro state. *Acta Trop.* 191 239–242. 10.1016/j.actatropica.2019.01.003 30615856

[B18] Guo W. P. Huang B. Zhao Q. Xu G. Liu B. Wang Y. H. (2018). Human-pathogenic *Anaplasma* spp., and Rickettsia spp. in animals in Xi’an. China. *PLoS Negl. Trop. Dis.* 12:e0006916. 10.1371/journal.pntd.0006916 30419024 PMC6258427

[B19] Halajian A. Palomar A. M. Portillo A. Heyne H. Romero L. Oteo J. A. (2018). Detection of zoonotic agents and a new *Rickettsia* strain in ticks from donkeys from South Africa: implications for travel medicine. *Travel. Med. Infect. Dis.* 26 43–50. 10.1016/j.tmaid.2018.10.007 30312734

[B20] Han R. Yang J. F. Mukhtar M. U. Chen Z. Niu Q. L. Lin Y. Q. (2019). Molecular detection of *Anaplasma* infections in ixodid ticks from the qinghai-tibet plateau. *Infect. Dis Poverty.* 8:12. 10.1186/s40249-019-0522-z 30728069 PMC6366118

[B21] Hao X. Liu R. He Y. Xiao X. Xiao W. Zheng Q. (2019). Multiplex PCR methods for detection of several viruses associated with canine respiratory and enteric diseases. *PLoS One.* 14:e0213295. 10.1371/journal.pone.0213295 30830947 PMC6398926

[B22] Henegariu O. Heerema N. A. Dlouhy S. R. Vance G. H. Vogt P. H. (1997). Multiplex PCR: critical parameters and step-by-step protocol. *Biotechniques* 23 504–511. 10.2144/97233rr01 9298224

[B23] Hornok S. De L. F. J. Biró N. Ig F. D. M. Meli M. L. Elek V. (2011). First molecular evidence of *Anaplasma ovis* and *Rickettsia* spp. in keds (*Diptera*: *Hippoboscidae*) of sheep and wild ruminants. *Vector Borne Zoonotic Dis.* 11 1319–1321. 10.1089/vbz.2011.0649 21923269

[B24] Hornok S. Szoke K. Meli M. L. Sandor A. D. Gorfol T. Estok P. (2019). Molecular detection of vector-borne bacteria in bat ticks (*Acari*: *Ixodidae*, *Argasidae*) from eight countries of the Old and New Worlds. *Parasit. Vectors* 12:50. 10.1186/s13071-019-3303-4 30670048 PMC6343265

[B25] Kawahara M. Rikihisa Y. Lin Q. Isogai E. Tahara K. Itagaki A. (2006). Novel genetic variants of *Anaplasma phagocytophilum*, *Anaplasma bovis*, *Anaplasma* centrale, and a novel Ehrlichia sp. in wild deer and ticks on two major islands in Japan. *Appl. Environ. Microbiol.* 72 1102–1109. 10.1128/AEM.72.2.1102-1109.2006 16461655 PMC1392898

[B26] Kim K. Yi J. Oh W. Kim N. Choi S. Choe P. (2014). Human granulocytic anaplasmosis, South Korea, 2013. *Emerging Infect Dis.* 20 1708–1711. 10.3201/eid2010.131680 25271737 PMC4193166

[B27] Kundave V. R. Ram H. Banerjee P. S. Garg R. Mahendran K. Ravikumar G. (2018). Development of multiplex PCR assay for concurrent detection of tick borne haemoparasitic infections in bovines. *Acta Parasitol.* 63 759–765. 10.1515/ap-2018-0090 30367760

[B28] Lee S. H. Park S. Lee Y. S. Lee H. K. Hwang S. D. (2018). Diagnosis and molecular characteristics of human infections caused by *Anaplasma phagocytophilum* in South Korea. *J. Microbiol.* 56 847–853. 10.1007/s12275-018-8385-8 30353471

[B29] Lew A. E. Gale K. R. Minchin C. M. Shkap V. Waal D. T. D. (2003). Phylogenetic analysis of the erythrocytic *Anaplasma* species based on 16S rDNA and GroEL (HSP60) sequences of A. marginale, A. centrale, and A. ovis and the specific detection of A. centrale vaccine strain. *Vet. Microbiol.* 92 145–160. 10.1016/S0378-1135(02)00352-8 12488078

[B30] Li H. Zheng Y. C. Ma L. Jia N. Jiang B. G. Jiang R. R. (2015). Human infection with a novel tick-borne *Anaplasma* species in China: a surveillance study. *Lancet Infect. Dis.* 15 663–670. 10.1016/S1473-3099(15)70051-4 25833289

[B31] Liu Z. Ma M. Wang Z. Wang J. Peng Y. Li Y. (2012). Molecular survey and genetic identification of *Anaplasma* species in goats from central and southern China. *Appl. Environ. Microbiol.* 78 464–470. 10.1128/AEM.06848-11 22057867 PMC3255723

[B32] Liu Z. Peasley A. M. Yang J. Li Y. Guan G. Luo J. (2019). The *Anaplasma ovis* genome reveals a high proportion of pseudogenes. *BMC Genomics* 20:69. 10.1186/s12864-018-5374-6 30665414 PMC6341658

[B33] M’ghirbi Y. Beji M. Oporto B. Khrouf F. Hurtado A. Bouattour A. (2016). Anaplasma marginale and A. phagocytophilum in cattle in Tunisia. *Parasit. Vectors*. 9:556. 10.1186/s13071-016-1840-7 27765073 PMC5072335

[B34] Peng Y. Wang K. Zhao S. Yan Y. Wang H. Jing J. (2018). Detection and Phylogenetic Characterization of *Anaplasma capra*: an emerging pathogen in sheep and Goats in China. *Front. Cell Infect. Microbiol.* 8:283. 10.3389/fcimb.2018.00283 30214896 PMC6126426

[B35] Pradeep R. K. Nimisha M. Sruthi M. K. Vidya P. Amrutha B. M. Kurbet P. S. (2019). Molecular characterization of South Indian field isolates of bovine *Babesia* spp. and *Anaplasma* spp. *Parasitol. Res.* 118 617–630. 10.1007/s00436-018-6172-4 30560519

[B36] Rjeibi M. R. Ayadi O. Rekik M. Gharbi M. (2018). Molecular survey and genetic characterization of *Anaplasma* centrale. A. marginale and A. bovis in cattle from Algeria. *Transbound Emerg Dis*. 65 456–464. 10.1111/tbed.12725 29034616

[B37] Seo M. G. Ouh I. O. Kwon O. D. Kwak D. (2018a). Molecular detection of *Anaplasma phagocytophilum*-like *Anaplasma* spp. and pathogenic *A. phagocytophilum* in cattle from South Korea. *Mol. Phylogenet Evol.* 126 23–30. 10.1016/j.ympev.2018.04.012 29653174

[B38] Seo M. G. Ouh I. O. Lee H. Geraldino P. J. L. Rhee M. H. Kwon O. D. (2018b). Differential identification of *Anaplasma* in cattle and potential of cattle to serve as reservoirs of *Anaplasma capra*, an emerging tick-borne zoonotic pathogen. *Vet. Microbiol.* 226 15–22. 10.1016/j.vetmic.2018.10.008 30389039

[B39] Song R. Wang Q. Guo F. Liu X. Song S. Chen C. (2018). Detection of *Babesia* spp., *Theileria* spp. and *Anaplasma ovis* in Border Regions, northwestern China. *Transbound Emerg Dis.* 65 1537–1544. 10.1111/tbed.12894 29932497

[B40] Sun X. F. Zhao L. Wen H. L. Luo L. M. Yu X. J. (2015). *Anaplasma* species in China. *Lancet Infect. Dis.* 15 1263–1264. 10.1016/S1473-3099(15)00377-1 26531037

[B41] Torina A. Agnone A. Blanda V. Alongi A. D’Agostino R. Caracappa S. (2012). Development and validation of two PCR tests for the detection of and differentiation between *Anaplasma ovis* and *Anaplasma marginale*. *Ticks Tick Borne Dis.* 3 283–287. 10.1016/j.ttbdis.2012.10.033 23182548

[B42] Wang J. Zhang Y. Cui Y. Yan Y. Wang X. Wang R. (2018). A rapid, simple and sensitive loop-mediated isothermal amplification method to detect *Anaplasma bovis* in sheep and goats samples. *Parasitol. Int.* 67 70–73. 10.1016/j.parint.2017.03.005 28351721

[B43] Wei R. Liu H. B. Jongejan F. Jiang B. G. Chang Q. C. Fu X. (2017). Cultivation of *Anaplasma ovis* in the HL-60 human promyelocytic leukemia cell line. *Emerg. Microbes Infect.* 6:e83. 10.1038/emi.2017.70 28928415 PMC5625320

[B44] Yang B. Sun E. Wen Y. Ye C. Liu F. Jiang P. (2019). Molecular evidence of coinfection of *Anaplasma* species in small ruminants from Anhui Province. *China. Parasitol. Int.* 71 143–146. 10.1016/j.parint.2019.04.004 30991112

[B45] Yang J. Han R. Niu Q. Liu Z. Guan G. Liu G. (2018a). Occurrence of four *Anaplasma* species with veterinary and public health significance in sheep, northwestern China. *Ticks Tick Borne Dis.* 9 82–85. 10.1016/j.ttbdis.2017.10.005 29037826

[B46] Yang J. Liu Z. Niu Q. Mukhtar M. U. Guan G. Liu G. (2018b). A novel genotype of “*Anaplasma capra*” in wildlife and its phylogenetic relationship with the human genotypes. *Emerg. Microbes Infect.* 7:210. 10.1038/s41426-018-0212-0 30538218 PMC6290010

[B47] Yang J. Liu Z. Niu Q. Liu J. Han R. Liu G. (2016). Molecular survey and characterization of a novel *Anaplasma* species closely related to *Anaplasma capra* in ticks, northwestern China. *Parasit Vectors.* 9:603. 10.1186/s13071-016-1886-6 27884197 PMC5123347

[B48] Zaid T. Ereqat S. Nasereddin A. Al-Jawabreh A. Abdelkader A. Abdeen Z. (2019). Molecular characterization of *Anaplasma* and *Ehrlichia* in ixodid ticks and reservoir hosts from Palestine: a pilot survey. *Vet. Med. Sci.* 5 230–242. 10.1002/vms3.150 30762295 PMC6498520

[B49] Zhou Z. Wu Y. Chen Y. Wang Z. Hu S. Zhou R. (2018). Molecular and serological prevalence of *Toxoplasma gondii* and *Anaplasma* spp. infection in goats from Chongqing Municipality. *China. Parasite.* 25 20. 10.1051/parasite/2018024 29633708 PMC5892175

[B50] Zhuo M. Calev H. Saunders S. J. Li J. Stillman I. E. Danziger J. (2019). Acute kidney injury associated with human granulocytic *Anaplasmosis*: a case report. *Am. J. Kidney Dis.* 74 696–699. 10.1053/j.ajkd.2019.03.428 31200977

