# Supplementary material for: A Multiplex PCR Detection Assay for the Identification of Clinically Relevant Anaplasma Species in Field Blood Samples
Source: Front Microbiol. 2020 Apr 7;11:606. doi: 10.3389/fmicb.2020.00606 (PMC7154085; doi:10.3389/fmicb.2020.00606)
Supplement: Supplementary file 1 [file Table_1.DOCX]

**Appendix**

Reproducibility of Multiplex PCR for detecting two clinical samples coinfection with four *Anaplasma* atgens

|  | | | *A.capra* | | | | *A. bovis* | | | | *A. ovis* | | | | *A. phagocytophilum* | | | |
| --- | --- | --- | --- | --- | --- | --- | --- | --- | --- | --- | --- | --- | --- | --- | --- | --- | --- | --- |
|  |  |  | 10^0^ | 10^-1^ | 10^-2^ | 10^-3^ | 10^0^ | 10^-1^ | 10^-2^ | 10^-3^ | 10^0^ | 10^-1^ | 10^-2^ | 10^-3^ | 10^0^ | 10^-1^ | 10^-2^ | 10^-3^ |
| Sample A | Inter-assay | Intra-assay | 1 | 1 | 1 | 0 | 1 | 1 | 1 | 0 | 1 | 1 | 0 | 0 | 1 | 1 | 1 | 1 |
|  |  |  | 1 | 1 | 0 | 0 | 1 | 1 | 1 | 0 | 1 | 1 | 1 | 0 | 1 | 1 | 1 | 0 |
|  |  |  | 1 | 1 | 1 | 0 | 1 | 1 | 1 | 0 | 1 | 1 | 0 | 0 | 1 | 1 | 1 | 0 |
|  |  | Intra-assay | 1 | 1 | 1 | 0 | 1 | 1 | 1 | 0 | 1 | 1 | 1 | 0 | 1 | 1 | 1 | 0 |
|  |  |  | 1 | 1 | 1 | 0 | 1 | 1 | 1 | 0 | 1 | 1 | 1 | 0 | 1 | 1 | 1 | 0 |
|  |  |  | 1 | 1 | 0 | 0 | 1 | 1 | 1 | 0 | 1 | 1 | 1 | 0 | 1 | 1 | 1 | 0 |
|  |  | Intra-assay | 1 | 1 | 1 | 0 | 1 | 1 | 1 | 0 | 1 | 1 | 1 | 0 | 1 | 1 | 1 | 0 |
|  |  |  | 1 | 1 | 1 | 0 | 1 | 1 | 1 | 0 | 1 | 1 | 1 | 0 | 1 | 1 | 1 | 0 |
|  |  |  | 1 | 1 | 0 | 0 | 1 | 1 | 0 | 0 | 1 | 1 | 1 | 0 | 1 | 1 | 1 | 0 |
|  | Kendall’s W | | 0.800, p<0.001 | | | | 0.905, p<0.001 | | | | 0.839, p<0.001 | | | | 0.889, p<0.001 | | | |
| Sample B | Inter-assay | Intra-assay | 1 | 1 | 0 | 0 | 1 | 1 | 1 | 0 | 1 | 1 | 0 | 0 | 1 | 1 | 1 | 0 |
|  |  |  | 1 | 1 | 0 | 0 | 1 | 1 | 1 | 0 | 1 | 1 | 1 | 0 | 1 | 1 | 0 | 0 |
|  |  |  | 1 | 1 | 0 | 0 | 1 | 1 | 0 | 0 | 1 | 0 | 1 | 0 | 1 | 1 | 1 | 0 |
|  |  | Intra-assay | 1 | 1 | 0 | 0 | 1 | 1 | 1 | 0 | 1 | 1 | 1 | 0 | 1 | 1 | 1 | 0 |
|  |  |  | 1 | 1 | 0 | 0 | 1 | 1 | 0 | 0 | 1 | 1 | 1 | 0 | 1 | 1 | 1 | 0 |
|  |  |  | 1 | 1 | 0 | 0 | 1 | 1 | 1 | 0 | 1 | 1 | 1 | 0 | 1 | 1 | 1 | 0 |
|  |  | Intra-assay | 1 | 0 | 1 | 0 | 1 | 1 | 0 | 0 | 1 | 1 | 1 | 0 | 1 | 1 | 0 | 0 |
|  |  |  | 1 | 1 | 0 | 0 | 1 | 1 | 1 | 0 | 1 | 1 | 1 | 0 | 1 | 1 | 1 | 0 |
|  |  |  | 1 | 1 | 0 | 0 | 1 | 1 | 1 | 0 | 1 | 1 | 1 | 0 | 1 | 1 | 1 | 0 |
|  | Kendall’s W | | 0.802, p<0.001 | | | | 0.800, p<0.001 | | | | 0.808, p<0.001 | | | | 0.839, p<0.001 | | | |

1 stands for positive, 0 stands for negative
